# Elevated expression of *RAB3B* plays important roles in chemoresistance and metastatic potential of hepatoma cells

**DOI:** 10.1186/s12885-022-09370-1

**Published:** 2022-03-11

**Authors:** Ryouichi Tsunedomi, Kiyoshi Yoshimura, Yuta Kimura, Mitsuo Nishiyama, Nobuyuki Fujiwara, Satoshi Matsukuma, Shinsuke Kanekiyo, Hiroto Matsui, Yoshitaro Shindo, Yusaku Watanabe, Yukio Tokumitsu, Shin Yoshida, Michihisa Iida, Nobuaki Suzuki, Shigeru Takeda, Tatsuya Ioka, Shoichi Hazama, Hiroaki Nagano

**Affiliations:** 1 Department of Gastroenterological, Breast and Endocrine Surgery, Yamaguchi University Graduate School of Medicine, 1-1-1 Minami-Kogushi, Ube, Yamaguchi 755-8505 Japan; 2 Department of Clinical Research in Tumor Immunology, Showa University Clinical Research Institute for Clinical Pharmacology and Therapeutics, Shinagawa, Tokyo 142-8555 Japan; 3 Oncology Center, Yamaguchi University Hospital, Ube, Yamaguchi 755-8505 Japan; 4 Department of Translational Research and Developmental Therapeutics against Cancer, Yamaguchi University Faculty of Medicine, Ube, Yamaguchi 755-8505 Japan

**Keywords:** Cancer stem cell, Hepatoma, RAB3B, Exosome, Sphere

## Abstract

**Background:**

Cancer stem cells (CSCs) are thought to play important roles in carcinogenesis, recurrence, metastasis, and therapy-resistance. We have successfully induced cancer stem-like sphere cells (CSLCs) which possess enhanced chemoresistance and metastatic potential. To enable the development of targeted therapy against CSLCs, we identified a gene responsible for this phenotype in CSLC.

**Methods:**

Human hepatoma cell line SK-HEP-1 was used for CSLC induction with a unique sphere inducing medium, and HuH-7 cells were used as non-sphere forming cells in the same condition. RNA-sequencing was performed followed by validation with quantitative RT-PCR and western blotting. Knockdown experiments were done by using CRISPR-Cas9 genome-editing, and the rescue experiments were performed using the expressing plasmid vector. Chemoresistance and liver metastasis of the cells, was studied following the splenic injection of cells to severely immune deficient mice and evaluated using the MTS assay. Quantification of exosomes in the medium was done using ELISA.

**Results:**

*RAB3B* was identified as an up-regulated gene in both CSLCs and prognostically poor hepatocellular carcinoma (HCC) by RNA-sequencing. *RAB3B*-KD cells showed altered CSLC phenotypes such as sphere formation, chemoresistance, and metastatic potentials, and those were rescued by *RAB3B* complementation. Increased exosome secretion was observed in CSLCs, and it was not observed in the *RAB3B*-KD cells. In addition, the *RAB3B* expression correlated with the expression of *ABCG2*, *APOE*, *LEPR*, *LXN*, and *TSPAN13*.

**Conclusion:**

The up regulation of *RAB3B* may play an important role in the chemoresistance and metastatic potential of CSLCs.

**Supplementary Information:**

The online version contains supplementary material available at 10.1186/s12885-022-09370-1.

## Background

Hepatocellular carcinoma (HCC) is among the most common cancers occurring worldwide, and it has a poor prognosis owing to a high recurrence rate [1]. Most potentially curative therapies for HCC, such as surgical resection, transplantation, and ablation therapy, have limited efficacy in advanced stages, and metastatic recurrence or de novo development of HCC occurs in approximately 70% of these patients within 5 years [2–6]. Postoperative recurrence is the leading cause of death in these patients [7, 8] which typically occurs within 2 years of resection [9, 10]. The benefits of adjuvant therapy have not been definitively demonstrated for various types of postoperative therapies following curative treatment.

Cancer stem cells (CSCs) are a small subset of cancer cells within the tumor bulk that are potentially responsible for malignant properties of tumors, such as tumor initiation, metastasis, recurrence, and chemoresistance [11–14]. They are produced via the accumulation of mutations in normal stem cells. In contrast, cancer cells differentiated from CSCs acquire stem cell-like properties via epithelial-mesenchymal transition (EMT) thereby behaving like cancer stem-like cells (CSLCs) [15–18]. Owing to the plasticity of cancer, we successfully induced the formation of CSLCs from cell lines derived from human hepatoma and pancreatic cancers using a unique medium supplemented with neural survival factor-1 (NSF-1) [16, 17]. The obtained CSLC spheres exhibit increased resistance to several anticancer drugs [16], are metastatic [18], and have increased expression of the EMT-related gene set [18]. Since the sphere cells, also called spheroids, have a three-dimensional (3D) structure, their cellular environment bears a closer resemblance to in vivo tumor conditions in comparison to conventional two-dimensional (2D) cell cultures. The CSLCs generated by us exhibited CD133^−^/CD44^high^/CD24^low^ expression unlike typical liver CSCs [16].

This study explored the genes responsible for the CSLC phenotype and poor prognosis of hepatocellular carcinoma (HCCs) using RNA-sequencing (RNA-seq) of several cell line derivatives and resected human specimens. Furthermore, we investigated the role of an interesting gene, *RAB3B*, in sphere formation, drug resistance, and metastatic potential of cells by performing knockdown (KD) and rescue experiments. *RAB3B* is one of the low-molecular-weight GTP-binding proteins (small G proteins) in the Rab family and acts as a central regulator of vesicular traffic [19]. Although the role of *RAB3B* in cancers is largely unknown, urinary exosomes in patients with prostate cancer reportedly contain high amounts of this protein [20]. Exosomes play an important role in the malignant transformation of cancer, including metastasis, through its contents such as microRNAs and proteins [21]. We also studied the effect of exosomes on CSLCs in this study.

## Methods

### Cell lines

SK-HEP-1 and HuH-7 cell lines, derived from human hepatoma, were purchased from the American Type Culture Collection (ATCC) (Rockville, MD, USA) and the Health Science Research Resources Bank (Osaka, Japan), respectively. Cells were cultured in Dulbecco’s modified Eagle’s medium (DMEM; Nissui Pharmaceutical, Tokyo, Japan) containing 10% heat-inactivated fetal bovine serum (Thermo Fisher Scientific, Kanagawa, Japan), penicillin (100 U/mL), streptomycin (100 μg/mL), and sodium bicarbonate (1.5 g/L) at 37 °C in a humidified atmosphere with 5% CO_2_ in air.

### Patients

Samples were obtained with written informed consent from 14 patients who underwent curative hepatectomy for HCC between August 2002 and November 2007 in the Department of Digestive Surgery and Surgical Oncology, Yamaguchi University Graduate School of Medicine, Japan. The study protocol conformed to the ethical guidelines of the 1975 Declaration of Helsinki as reflected in a prior approval by the Institutional Review Board for Human Use at Yamaguchi University Graduate School of Medicine. Ten samples each were used for RNA-seq and quantitative real-time polymerase chain reaction (qRT-PCR); six samples were common to both the analyses.

### Induction of sphere cells

Cells were suspended in the sphere inducing medium, which was based on a neural stem cell medium [16]. This medium, used to induce floating sphere cells, was DMEM/Nutrient Mixture F-12 Ham supplemented with 0.6% glucose, 10 mM HEPES, 2 μg/mL heparin, 0.1 mg/mL transferrin, 25 μg/mL insulin, 60 μM putrescine, 30 nM sodium selenite, 20 nM progesterone, 10 ng/mL human recombinant epidermal growth factor (all from Sigma-Aldrich Japan, Tokyo, Japan), 10 ng/mL basic fibroblast growth factor (Merck Millipore, Tokyo, Japan), 10 ng/mL leukemia inhibitory factor (Merck Millipore), 60 μg/mL N-acetyl-L-cysteine (Sigma-Aldrich), and 1/50 volume NSF-1 (Lonza, Tokyo, Japan).

### RNA-sequencing

Total RNA was isolated with the miRNeasy Mini Kit (Qiagen, Tokyo, Japan). Sequencing libraries were constructed using the TruSeq Stranded Total RNA with Ribo-Zero Gold LT Sample Prep kit (Illumina, Tokyo, Japan) according to the manufacturer’s instructions. Sequencing of paired-end fragments (75 bp × 2) was conducted on a NextSeq 500 sequencing platform (Illumina).

After a quality control step, the filtered short reads were mapped to the reference genome (hg38) with STAR (version 2.5.1b) [22]. Strand-specific counts of fragments from each sample were obtained using RSEM (version 1.3.3) [23] and normalized with the trimmed mean of M-values method [24] using the TCC package [25, 26]. The edgeR (version 3.28.1) [27, 28] package was used to identify the differentially expressed genes (DEGs) based on a false discovery rate *q*-value threshold < 0.05.

### Quantitative real-time polymerase chain reaction (qRT-PCR)

The mRNA expression was examined by qRT-PCR as described previously [10]. qRT-PCR was performed using a LightCycler 480 Probe Master (Roche Diagnostics, Tokyo, Japan) and Universal ProbeLibrary (Roche Diagnostics) probes or a LightCycler 480 SYBR Green I Master (Roche Diagnostics) on a LightCycler 480 System II (Roche Diagnostics). The primers and probes used are listed in Supplementary Table S1. Amplification was performed in a two-step procedure and mRNA levels were measured quantitatively using the Δ/Δ threshold cycle method. Glyceraldehyde-3-phosphate dehydrogenase (*GAPDH*) and phosphoglycerate kinase 1 (*PGK1*) were used as controls. Triplicate wells were analyzed for each assay.

### Western blot analysis

Cells were lysed and the proteins (10 μg) were separated by SDS-PAGE on an 8% gel and transferred onto a Poly (vinylidene fluoride) (PVDF) membrane (Bio-Rad, Tokyo, Japan) as described previously [29]. Membranes were blocked with 3% skim milk and treated with the primary antibodies, anti-RAB3B (ab55655; Abcam, Tokyo, Japan) and anti-VCP (anti-valosin-containing protein), (GTX113030, GeneTex, Alton Pkwy Irvine, CA, USA). The immunoreactive bands were visualized using an ECL Pro (PerkinElmer, Waltham, MA) and Amersham Imager (GE Healthcare, Tokyo, Japan), and quantified using the ImageJ software (National Institutes of Health, USA). VCP was used as the loading control because its levels are more stable compared to those of other loading controls, such as GAPDH and β-actin [29].

### Genome editing for *RAB3B*

A guide RNA (gRNA) targeting a sequence in *RAB3B* (5′-GTTTCACCCGCTTCTCGTGA-3′) was constructed by in vitro transcription using a GeneArt Precision gRNA Synthesis Kit (Thermo Fisher Scientific) according to the manufacturer’s instructions (Supplementary Fig. S1a). The gRNA and Cas9 mRNA (GeneArt CRISPR Nuclease mRNA, Thermo Fisher Scientific) were transfected into cells using Lipofectamine MessengerMAX (Thermo Fisher Scientific). Genomic DNA (gDNA) from isolated monoclonal clones was subjected to Sanger sequencing (Supplementary Fig. S1b). The *RAB3B*-edited clone derived from SK-HEP-1 was named *RAB3B*-KD. The *RAB3B*-KD cells were transfected with pcDNA3.1(−) (Thermo Fisher Scientific) harboring full-length *RAB3B* cDNA, pRAB3B, using Lipofectamine 3000 (Thermo Fisher Scientific) to generate *RAB3B* rescued KD/pRAB3B cells. A full-length *RAB3B* cDNA (NM_002867.3: position 214 to 873) with Kozak, 5′-flanking EcoRI, and 3′-flanking BamHI sequences was synthesized by FASMAC (Kanagawa, Japan) and it was inserted into the site downstream of the CMV promoter of pcDNA3.1(−).

### Cell viability assay

The CellTiter 96 AQueous One Solution Cell Proliferation Assay (Promega, Tokyo, Japan), which includes a tetrazolium compound [3-(4,5-dimethylthiazol-2-yl)-5-(3-carboxymethoxyphenyl)-2-(4-sulfophenyl)-2H-tetrazolium, inner salt; MTS] was used according to the manufacturer’s instructions. Cells in culture medium were incubated with one of the following anticancer drugs, 10 mM 5-fluorouracil (5-FU, Sigma-Aldrich), 250 nM docetaxel (Sigma-Aldrich), 2 μM doxorubicin (Sigma-Aldrich), 200 μM irinotecan hydrochloride (Sigma-Aldrich), 2.5 μM suberoylanilide hydroxamic acid (SAHA, Cosmo Bio), 75 μM sorafenib tosylate (ChemScene, Monmouth Junction, NJ), 25 μM lenvatinib mesylate (Carbosynth, Berkshire, UK), 50 μM regorafenib (ChemScene), or 20 μM cabozantinib S-malate (ChemScene), 24 h at 37 °C in an atmosphere of 5% CO_2_ in air. Cells incubated in culture medium alone were used as control. The optical density of the culture medium at 492 and 650 nm was measured by using an EnVision plate reader (PerkinElmer). The viability of cells treated with the anticancer drugs was calculated as a ratio with respect to their viability in the absence of anticancer drugs. Triplicate wells were analyzed for each assay.

### Splenic injection of tumor cells

NOD-Rag1^null^ IL2rγ^null^ double mutant mice (NRG mice) were purchased from the Jackson Laboratory (Bar Harbor, ME, USA) and maintained in a HEPA-filtered environment with autoclave-sterilized cages, food, and bedding. All animal studies were conducted in accordance with the Institutional Animal Care and Use Committee of Yamaguchi University and conformed to the Guide for the Care, Use of Laboratory Animals published by the United States National Institutes of Health (Bethesda, MD, USA), and ARRIVE guidelines.

The ability of cells to produce tumor nodules in the liver was studied subsequent to their implantation into the spleen of 8–12-week-old NRG female mice as described previously [18]. After 8 weeks, injected mice were sacrificed and necropsied.

### Quantitative analysis of exosomes

Cells were incubated for 24 h, after which the exhausted culture medium was replaced with fresh culture medium. At the same time, cell viability was measured from replicate plates using MTS assay and it was used for normalization of exosome quantification. For quantification of exosomes, the conditioned medium was collected and analyzed using a CD9/CD63 Exosome ELISA Kit, Human (Cosmo Bio) according to the manufacturer’s instructions. Signals were detected using an EnVision plate reader (PerkinElmer). If necessary, GW4869 hydrochloride hydrate (Cayman Chemical, Ann Arbor, MI) was added as an exosome inhibitor on the next day of cell seeding. Triplicate wells were analyzed for each assay.

### Statistical analysis

Each experiment was repeated at least three times. Data are expressed as means ± standard deviation. Significant differences were evaluated by the Tukey–Kramer multiple comparison, paired *t*-test, or Fisher’s exact test, using the R version 3.6.3 software (the R project website, http://www.r-project.org/). A *P* value of < 0.05 was considered statistically significant.

## Results

### Screening of CSLC-specific mRNA expression

**Fig. 1:**
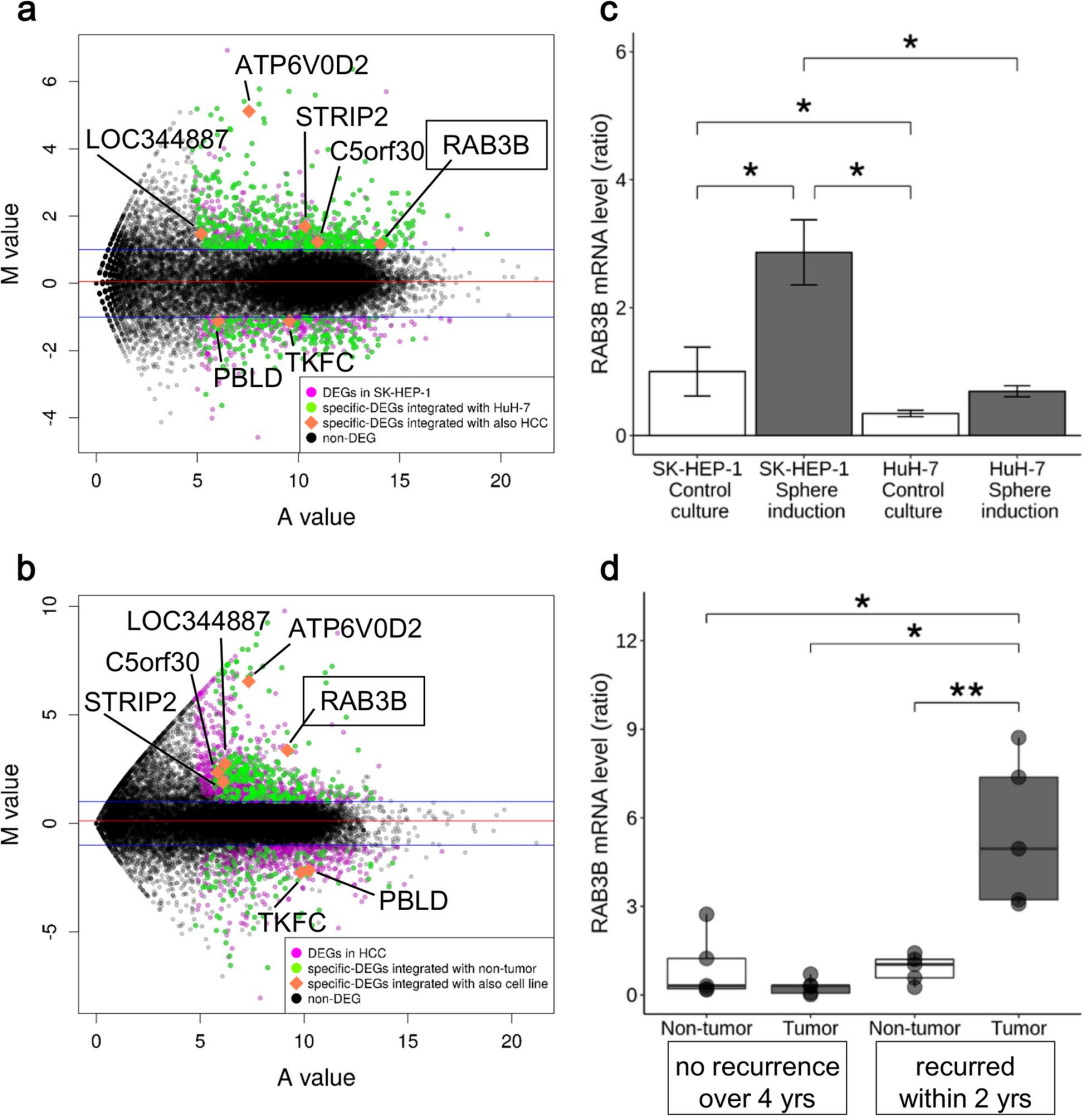
mRNA levels of *RAB3B*. **a** and **b**, M-A plots generated from RNA-sequence analysis. For each gene, the log_2_(average expression) in the two samples (A, *x* axis) against the log_2_(fold change) between the samples (M, *y* axis) is plotted. **a**, Magenta dots represent significant differentially expressed genes (DEGs) in SK-HEP-1 cells. Green dots indicate sphere-specific DEGs that are not significantly changed in the experiment using non-sphere forming HuH-7 cells. **b**, Magenta dots represent significant DEGs in primary HCCs with/without recurrence. Green dots also represent DEGs significantly changed in HCCs compared with non-tumorous liver tissue samples. Orange diamonds are overlapping DEGs. The mRNA levels of *RAB3B* in cell lines (**c**) and clinical specimens (**d**) were measured using qRT-PCR and are represented as the ratio of the expression with respect to that in SK-HEP-1 cells or non-tumorous liver tissues. Recurrence-free (*n* = 5) and recurrence (*n* = 5) cases consisting of a pair of HCC and adjacent non-cancerous liver samples were used. *P < 0.05 with Tukey–Kramer multiple comparison test. **, *P* < 0.05 with pairwise *t*-test

Using quantitative RNA-seq analysis, we compared the comprehensive mRNA levels in sphere-forming SK-HEP-1 and non-sphere-forming HuH-7 cells (Supplementary Fig. S3 left). In addition, mRNA levels in human HCC and surrounding liver tissue specimens were also compared. There were 1471 genes with significant differences in the counts between SK-HEP-1 cells in sphere inducing conditions and those in control culture (fold change > 2.0; *q* value < 0.05; magenta and green dots in Fig. 1a). Considering the mRNA levels in HuH-7 cells, with no sphere-forming potential [16], 755 genes were identified as specific DEGs in sphere-forming SK-HEP-1 cells (green dots in Fig. 1a).

To focus on clinically important genes, the DEGs were further screened with respect to recurrence after surgery (Supplementary Fig. S3 right). Two screening criteria were applied: one was the difference in expression between primary HCCs that recurred within 2 years after surgery and those without recurrence over 4 years, and the other was the difference in expression between HCCs and corresponding adjacent liver tissue. As a result, 435 genes were identified as DEGs regarding recurrence in HCC specimens (green dots in Fig. 1b).

Among the above DEGs, seven genes (*ATP6V0D2*, *C5orf30*, *LOC344887*, *PBLD*, *RAB3B*, *STRIP2*, and *TKFC*) were common in both the screenings with cell lines and HCC specimens (orange diamonds in Fig. 1a,b). We further focused on *RAB3B* because its mRNA levels were upregulated in sphere formation and were abundant in both cell lines and clinical samples (Supplementary Fig. S4). The expression of *RAB3B* mRNA under these conditions was validated using qRT-PCR (Fig. 1c,d).

### Knockdown of the CSLC-specific *RAB3B*

**Fig. 2:**
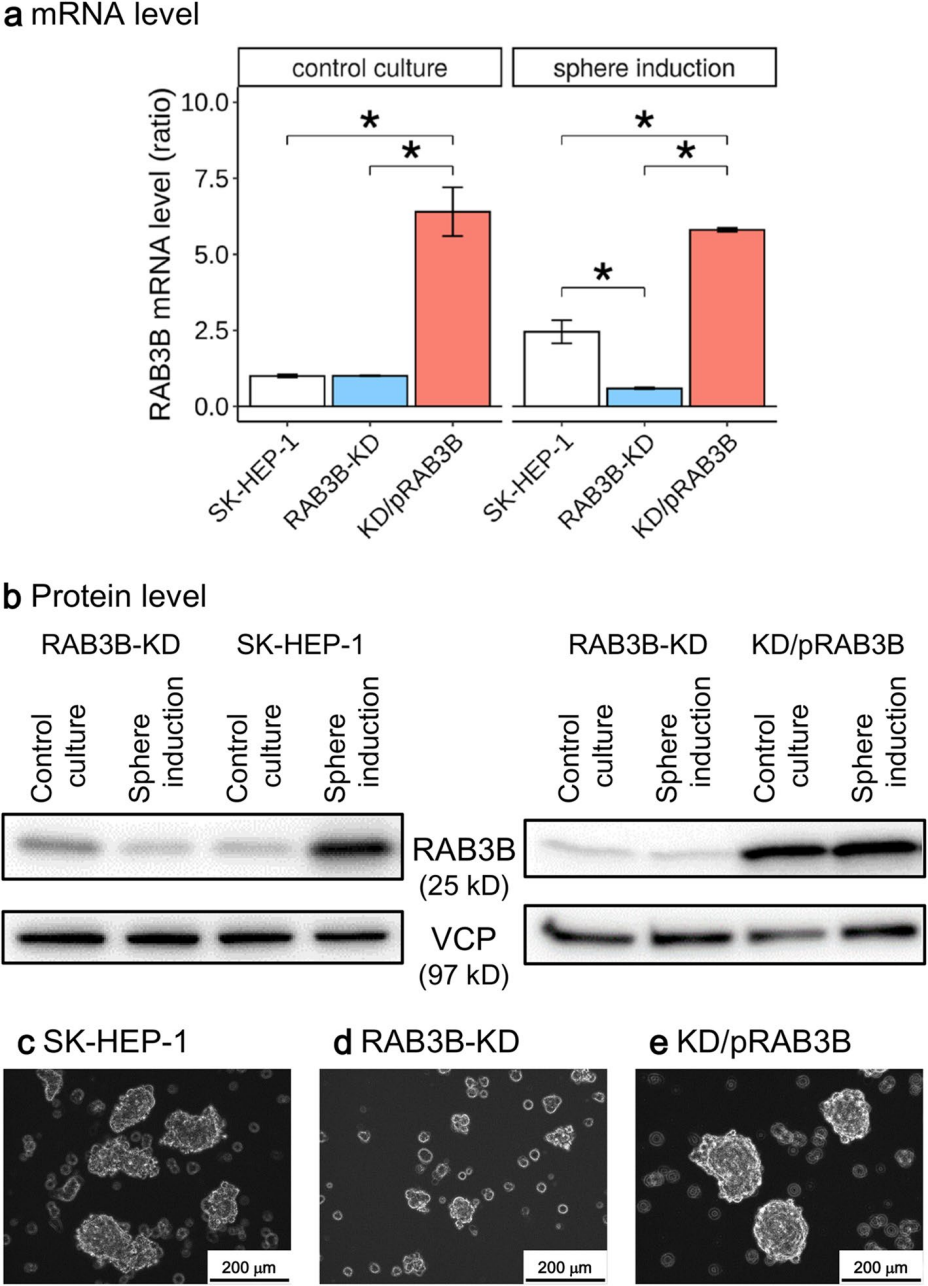
Expression of *RAB3B* and sphere formation ability of SK-HEP-1 derivative cells. **a**, RAB3B mRNA levels in SK-HEP-1 derivative cells. RAB3B mRNA levels were quantified using qRT-PCR, and the values were subsequently normalized as the mean ratio of the value from reference cells (SK-HEP-1 cells cultured in control medium). *P < 0.05 with Tukey–Kramer multiple comparison test. **b**, Western blot analysis of whole-cell lysates. The upper and lower immunoblottings were generated from the same gel, and the blots were separated around the indicated sizes and then reacted with each antibody, respectively. VCP was used as the loading control. **c–e**, Sphere cells derived from SK-HEP-1 (c), SK-HEP-1 harboring genome edited *RAB3B* (d), and the *RAB3B* edited SK-HEP-1 harboring a RAB3B expressing vector, pRAB3B (e)

We generated *RAB3B*-KD clones from SK-HEP-1 cells using the CRISPR/Cas9 system. We obtained a clone, which expressed truncated RAB3B, although this mutation was monoallelic (Supplementary Fig. S1, S2). The *RAB3B*-KD cells showed decreased *RAB3B* mRNA expression, even in the sphere inducing medium (0.3-fold, *P* < 0.01, Fig. 2a). Furthermore, the decreased *RAB3B* expression in *RAB3B*-KD cells was rescued by transfection with pRAB3B. Similarly, the induction of RAB3B in the sphere inducing medium diminished in the *RAB3B*-KD cells, and the plasmid vector rescued its expression (Fig. 2b). The antibody used for RAB3B recognized the region truncated by genome editing and therefore only RAB3B expressed from the non-mutated allele was detected in the western blot analysis.

Besides the reduced *RAB3B* expression, the *RAB3B*-KD cells showed insufficient sphere formation which was reinforced in the exogenous *RAB3B*-expressing *RAB3B*-KD cells (Fig. 2c–e). Similar to our observations in a previous study [16], SK-HEP-1 cells in the sphere inducing conditions showed a G0/G1 arrest (82.2% ± 0.4% in Supplementary Fig. S5b). In the sphere inducing conditions, *RAB3B*-KD cells showed a cell cycle distribution with 67.3% ± 0.5% cells in G0/G1, 3.6% ± 0.1% in S, and 29.1% ± 0.3% in G2/M phases (Supplementary Fig. S5d). This showed that an increased proportion of *RAB3B*-KD cells in the sphere inducing conditions were in G2/M phase compared to SK-HEP-1 cells in identical culture conditions. The cell cycle distribution of KD/pRAB3B cells in the sphere inducing condition was similar to that of SK-HEP-1 cells in identical conditions (Supplementary Fig. S5f). In control culture conditions, *RAB3B*-KD cells showed an increase in the S-phase population compared to SK-HEP-1 cells (Supplementary Fig. S5a,c).

### Effect of RAB3B expression on the susceptibility to anticancer drugs

**Fig. 3:**
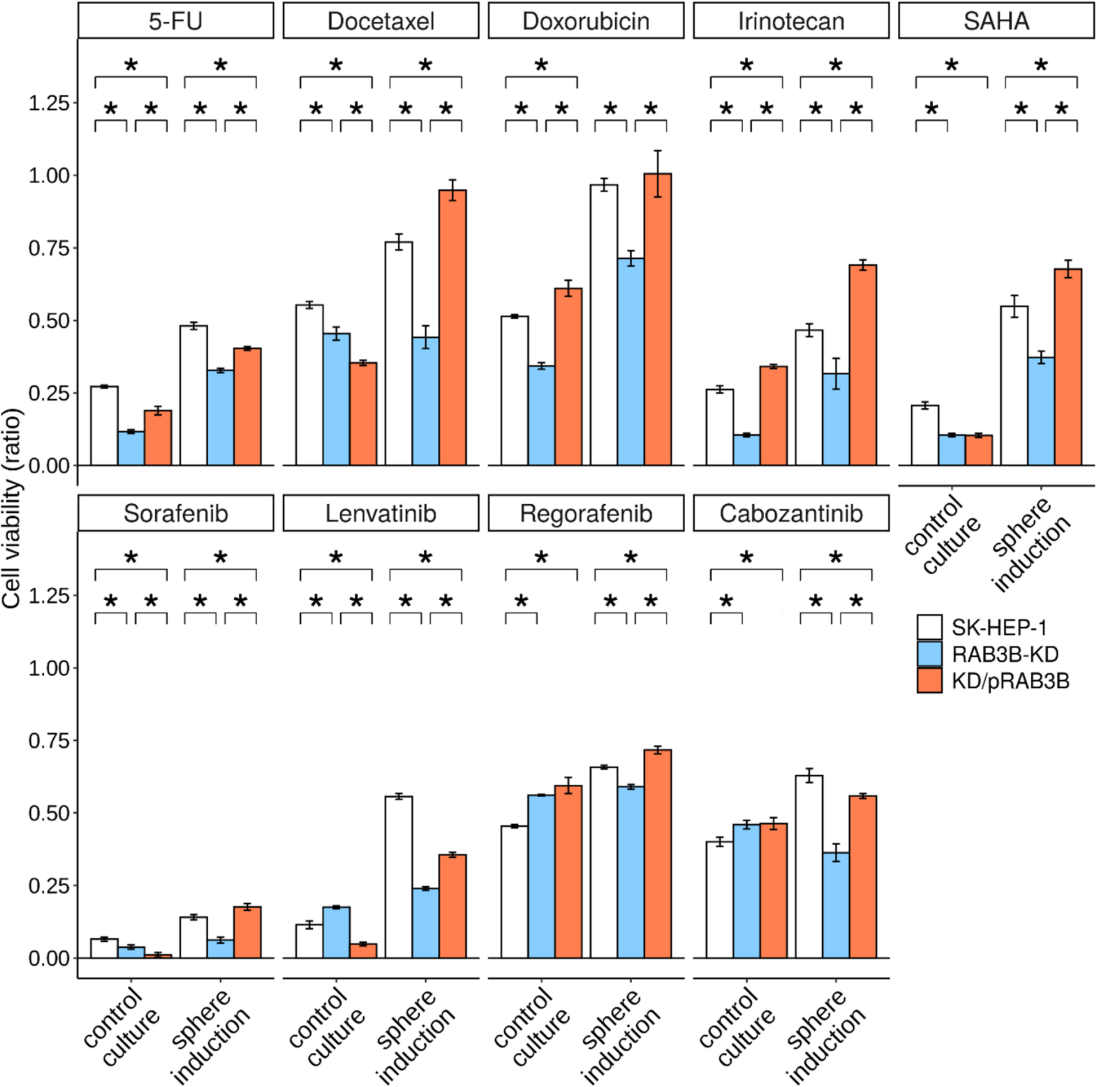
Susceptibility of SK-HEP-1 derivative cells to anticancer drugs. The viability of SK-HEP-1 (white columns), *RAB3B*-KD (blue columns), and KD/pRAB3B (salmon columns) cells in the presence of anticancer drugs (5-fluorouracil, docetaxel, doxorubicin, irinotecan, SAHA, sorafenib, lenvatinib, regorafenib, and cabozantinib) was evaluated using the MTS assay. For each anticancer drug, the left and right three columns represent data for cells in the control and sphere inducing cultures, respectively. *P < 0.05 with Tukey–Kramer multiple comparison test

In the sphere inducing conditions, SK-HEP-1 cells showed increased viability in the presence of the tested anticancer drugs compared to those in control conditions (Fig. 3). The *RAB3B*-KD cells showed decreased viability in the sphere inducing conditions in the presence of any of the tested drugs compared to the parental SK-HEP-1 and *RAB3B* rescued KD/pRAB3B cells. In the control condition, decreased and recovered viabilities of *RAB3B* engineered cells were observed in the presence of 5-FU, doxorubicin, and irinotecan.

### Ability of *RAB3B*-KD cells to metastasize to the liver

We examined the potential of *RAB3B*-KD cells to metastasize to the liver (Table 1, and Supplementary Fig. S6). In our previous study, injection of 1 × 10^3^ sphere cells into the spleen of NRG mice resulted in an increased frequency in the occurrence of liver tumors compared to the injection of the same number of parental SK-HEP-1 cells (50% vs. 14%, *P* < 0.05). On the contrary, *RAB3B*-KD cells subjected to sphere induction did not exhibit increased liver metastatic potential compared to cells cultured normally (17% vs. 19%).

**Table 1:** Liver metastasis ability of *RAB3B*-KD cells

	10^3^ cells inoculation				10^4^ cells inoculation			
	yes	no	(% of yes)	P*	yes	no	(% of yes)	P*
**SK-HEP-1**^**†**^								
Control	3	19	(14%)	0.018	7	3	(70%)	0.216
Sphere	9	9	(50%)		8	0	(100%)	
***RAB3B*-KD**								
Control	1	5	(17%)	1.000	6	3	(67%)	1.000
Sphere	4	20	(17%)		5	2	(71%)	

*, *P* values of Fisher’s exact test

^†^, the data of SK-HEP-1 is our previously published data [18]

### Quantification of exosomes released into the medium and their effect on CSLCs

**Fig. 4:**
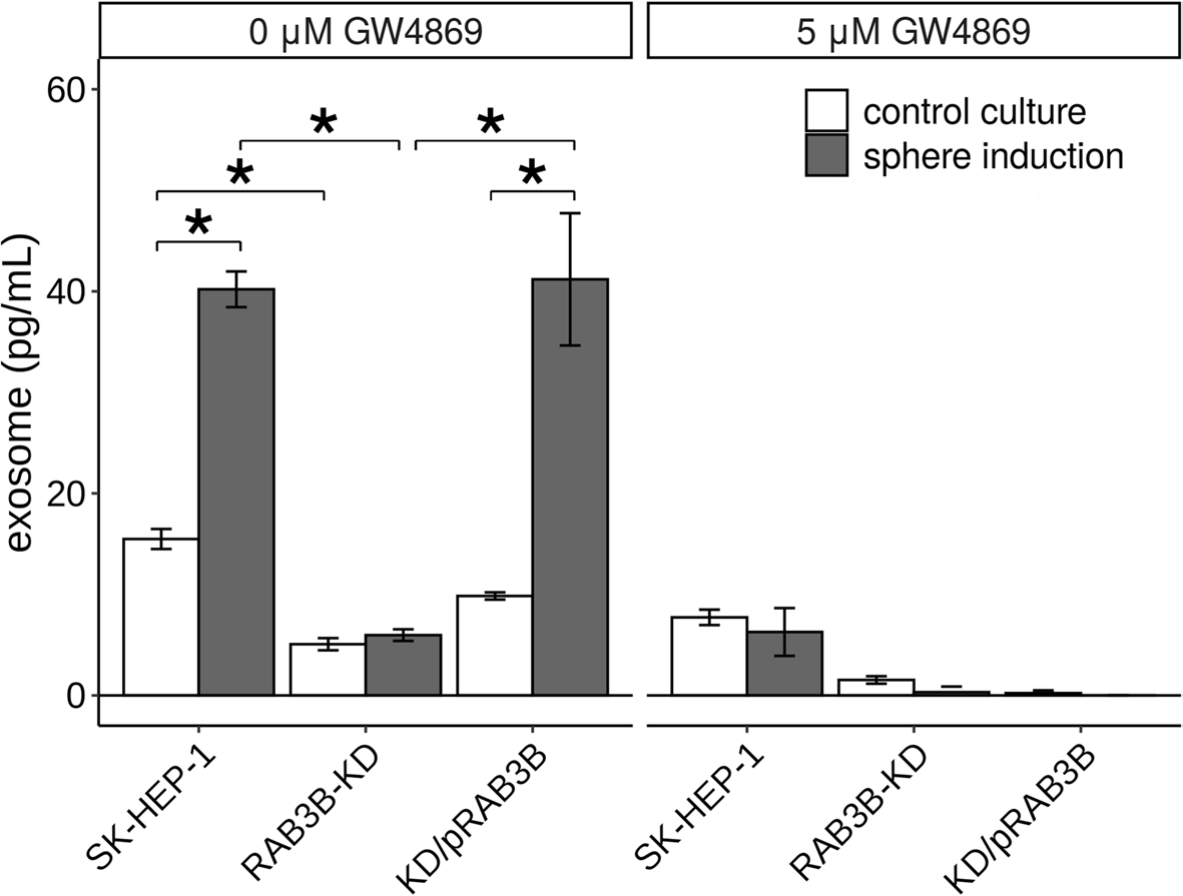
Exosome levels released from SK-HEP-1 derivatives. Quantification of exosomes. White and gray columns represent values from the control and sphere inducing medium, respectively. If necessary, the exosome inhibitor, GW4869 hydrochloride hydrate (final concentration, 5 μM), was added. *P < 0.05 with Tukey–Kramer multiple comparison test

**Fig. 5:**
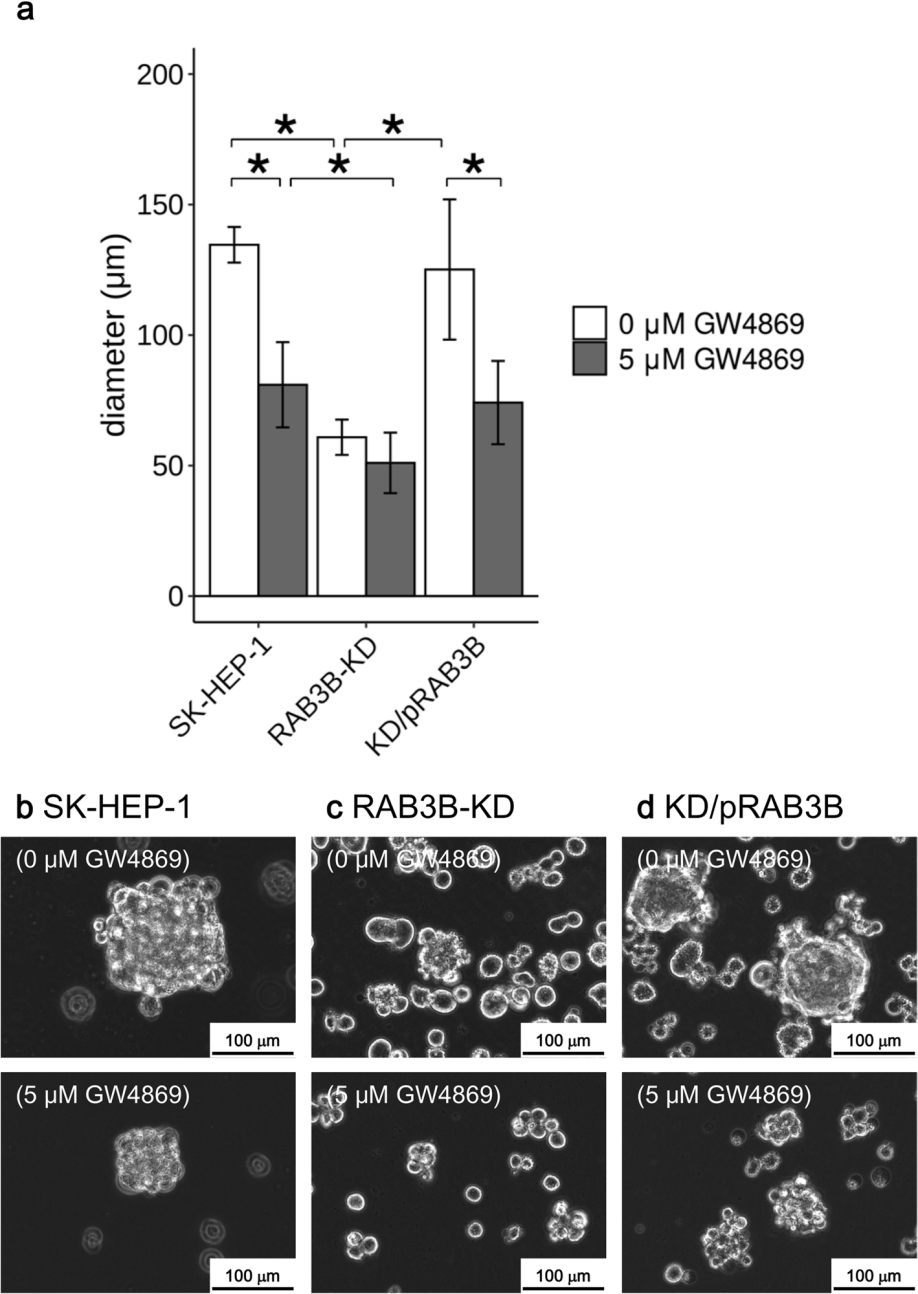
Effect of an exosome inhibitor on sphere formation of SK-HEP-1 derivative cells. **a**, Size of sphere cells. White and gray columns represent size of sphere cells in the absence and presence of the exosome inhibitor, GW4869 hydrochloride hydrate (final concentration, 5 μM). *P < 0.05 with Tukey–Kramer multiple comparison test. **b–d**, Representative sphere cells in the absence (upper panels) and presence (lower panels) of 5 μM GW4869

We examined the amount of exosomes in the medium because certain Rab families regulate the exocytosis of vesicles [30]. For SK-HEP-1 cells, the exosome level was significantly increased (2.6-fold, *P* < 0.01) in the sphere inducing medium compared to that in the control medium (Fig. 4). In the *RAB3B*-KD cells, the number of exosomes was not increased in the sphere inducing medium. The KD/pRAB3B cells also showed increased exosome release (4.2-fold, *P* < 0.01), although the number of exosomes released in the control medium was similar to that in the case of SK-HEP-1 cells. The addition of an exosome inhibitor, GW4869 (final concentration, 5 μM), repressed the exosome release in all the conditions tested (Fig. 4). The sphere size of SK-HEP-1 and KD/pRAB3B cells was significantly smaller (0.6-fold each, *P* < 0.05) in the presence of GW4869 than that in its absence (Fig. 5).

### Expression profile of genes affected by *RAB3B* expression

**Fig. 6:**
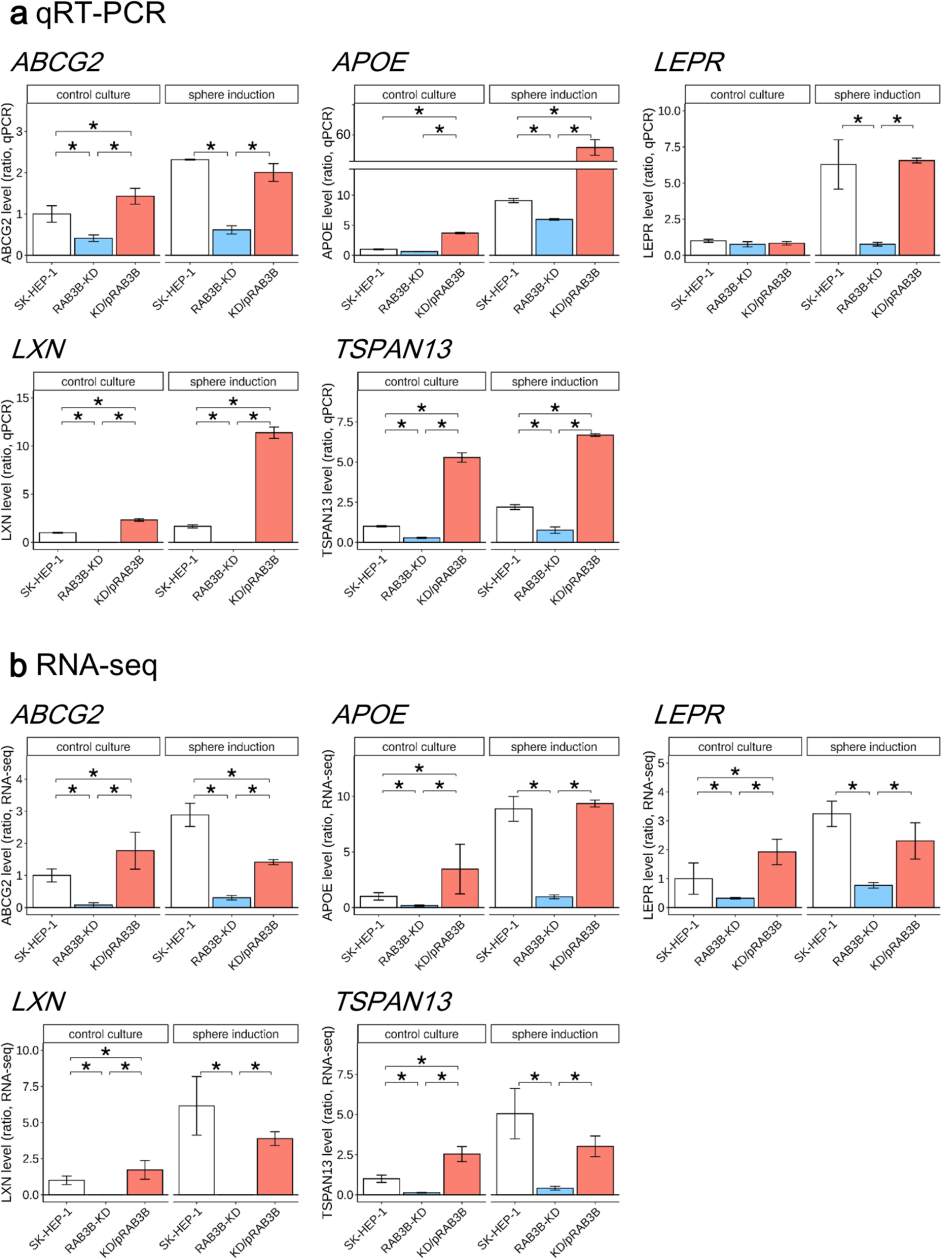
RAB3B-affected CSLC-specific gene expression. **a**, The five genes (*ABCG2*, *APOE*, *LEPR*, *LXN*, and *TSPAN13*) were validated as the RAB3B-affected CSLC-specific genes. The mRNA expression of each gene from SK-HEP-1 (white columns), *RAB3B*-KD (blue columns), and KD/pRAB3B (salmon columns) cells was evaluated using quantitative RT-PCR. *P < 0.05 with Tukey–Kramer multiple comparison test. **b**, The mRNA expressions counted by RNA-sequencing. *q < 0.05

To comprehensively investigate the effect of *RAB3B* expression on the CSLC phenotype, we performed RNA-seq analysis using *RAB3B*-KD and KD/pRAB3B cells in addition to the parental SK-HEP-1 cells (Supplementary Fig. S7). The screening was performed according to the following criteria: 1. changes in expression in the sphere inducing conditions compared to that in the control condition; 2. changes in expression in constitutive *RAB3B* over-expressing cells compared to SK-HEP-1 cells, and 3. changes in expression in the *RAB3B* knocked down condition in both sphere inducing and control conditions. From the screening, we obtained 13 genes that showed specific expression under the sphere inducing conditions and their expression was associated with that of *RAB3B*. Among 13 genes, 5 genes (*ABCG2*, *APOE*, *LEPR*, *LXN*, and *TSPAN13*) passed the validation analysis using qRT-PCR (Fig. 6). *ABCG2* was one of these 5 genes. The expression of *ABCG2* was increased (qRT-PCR; 2.3-fold, *P* < 0.05, RNA-seq; 2.9-fold, *q* < 0.01) in SK-HEP-1 cells in the sphere inducing conditions compared to that in the control culture. In contrast, the expression of *ABCG2* in *RAB3B*-KD cells in both sphere inducing and control conditions was lower (qRT-PCR; < 0.4-fold, *P* < 0.05, RNA-seq; < 0.1-folds, *q* < 0.01) than that in SK-HEP-1 cells. The decrease in expression was recovered in KD/pRAB3B cells. The trend of expression of *LXN* and *TSPAN13* was similar to that of *ABCG2*. The expression of *APOE*, *LEPR*, and *RAB3B* was significantly different in sphere inducing conditions but not in control conditions.

## Discussion

We performed comprehensive RNA expression profiling of HCC specimens with recurrence and a sphere-forming cell line, and detected seven significant DEGs in CSLCs (Fig. 1). Among these 7 genes, we focused on *RAB3B*, which was upregulated and relatively abundant in both sphere cells and HCCs with a shorter recurrence-free period (Supplementary Fig. S4). *RAB3B*-KD and its constitutive rescued cells were generated and used to examine the effects of *RAB3B* on the cancer stem-like phenotype. Interestingly, the *RAB3B*-KD cells exhibited a smaller sphere size and increased susceptibility to anticancer drugs compared to parental cells; these effects were reverted upon exogenous expression of *RAB3B* in the *RAB3B*-KD cells (Figs. 2, 3 and 5). With regards to the mechanism of *RAB3B* induction, it is known that the *RAB3B* is upregulated in the prostate via androgen receptor signaling [31], although our sphere inducing medium was not supplemented with androgen. In addition, several hormones other than androgen in the sphere inducing medium did not significantly affect sphere formation (data not shown). In this study, *RAB3B*-KD cells did not show the induction of *RAB3B* in the sphere inducing conditions despite the presence of the remaining wild-type allele (Fig. 2). The abovementioned regulation by androgen receptor signaling is subjected to a feed-forward regulatory loop with NKX3–1 [31]; therefore, *RAB3B* might also be involved in the loop regulation and its expression from monoallele may be insufficient for *RAB3B* induction. Interestingly, *RAB3B*-KD cells showed a 37% frequency of reads harboring genome-edited *RAB3B* by whole exome sequencing (Supplementary Fig. S2), while RNA-seq showed that the frequencies of those were only 2% in *RAB3B*-KD cells in both control and sphere inducing conditions (data not shown). Another possibility is that truncated RAB3B might inhibit wild-type RAB3B function. However, the mechanism of *RAB3B* induction remains unclear.

Interestingly, we observed that the RAB3B levels correlated with the exosome release (Fig. 4). Moreover, the effect of inhibition of the exosome release on the sphere forming ability of the cells was also observed. A well-known Rab family responsible for exosome secretion is RAB27 [30]. Exosomes play an important role in organotropic metastasis through its membrane integrins and contents [32, 33]. Indeed, no difference in the liver metastatic ability of *RAB3B*-KD cells prepared in control culture or under the sphere inducing condition was observed (Table 1). These observations suggest that RAB3B might be involved in the modulation of tumor microenvironment during metastasis. An exosome inhibitor, GW4869, used in this study, represses mature exosome releasing from multivesicular endosomes (MVEs) by inhibiting the inward budding of MVEs [34]. RAB27 plays a role in MVE docking at the plasma membrane to exosome secretion [30]. Similar to the role of RAB27 in exosome secretion, RAB3 is involved in synaptic vesicle exocytosis [19]. It is suggested that exosomes contain signals for cell-to-cell communication are released into the tumor microenvironment by RAB3B which might be necessary for acquiring the CSLC phenotype. However, we have not yet been able to elucidate the signals in detail in this study. A limitation of this study is that the detailed mechanism involved in RAB3B mediated acquisition of CSLC phenotype, in addition to the mechanism of *RAB3B* induction, have not been uncovered.

One well-known mechanism of chemoresistance is the upregulation of ABC transporters, which mediate the efflux of the anticancer drugs [35]. We previously reported that *ABCG2*, an ABC transporter, was upregulated in the sphere inducing conditions [16]. Herein, *ABCG2* was also identified as one of the specific genes associated with sphere formation and *RAB3B* expression (Fig. 6). In agreement with the results of our previous study, in the sphere inducing conditions, the proportion of SK-HEP-1 cells in the G0/G1 phase were increased as compared to that in the control condition (Supplementary Fig. S5). Although the proportion of *RAB3B*-rescued cells increased in the G0/G1 phase was the same as that of SK-HEP-1 cells, *RAB3B*-KD cells showed increased population in the G2/M phase in the sphere inducting conditions.

Furthermore, we found 5 genes that were associated positively with both sphere forming conditions and *RAB3B* expression (Fig. 6). In addition to *ABCG2*, *APOE* and *LFER* reportedly play a role in cancer stem cells [36, 37]. The expression of *TSPAN13* is also associated with poor prognosis of papillary thyroid cancer, pancreatic cancer, lung adenocarcinoma, and bladder cancer [38]. It is consistent with our results that *LXN* overexpression promotes cell cycle arrest at G0/G1 phase in SK-HEP-1 cells, while the same study reported that *LXN* overexpression suppresses cell viability, colony formation, and tumorigenesis [39]. These previous findings may support the mechanism of acquisition of CSLC properties by *RAB3B* function in extracellular vesicle production.

Among the 7 genes that were associated with both sphere induction and poor prognosis of HCC, the expression of *PBLD*, *STRIP2*, and *LOC344887* has also been correlated with poor prognosis of HCC, lung adenocarcinoma, and non-small cell lung cance [40, 41]. Moreover, these genes are associated with EMT and invasive potential of cells [40, 42, 43]. Recently, *ATP6V0D2*, a proton transporter, which showed the highest M value in our study (Fig. 1), was reported to be involved in oncogenic functions, such as migration, invasion, and EMT [44]. Therefore, in addition to *RAB3B*, these genes may also contribute to the acquisition of the CSLC phenotype.

In conclusion, *RAB3B* might be required for the acquisition of CSLC properties. *RAB3B* might be crucial in the secretion of extracellular vesicles, such as exosomes, and might regulate the expression of related genes; hence, further detailed investigations on cell–cell communication would disclose key genes that can be targeted for the therapy of hepatoma.

## Supplementary Information

**Additional file 1: Table S1.** Primers and hydrolysis probes used in this study. **Supplementary Figure S1.** Genome editing of *RAB3B*. In each panel, upper and lower figures show Sanger sequencing data and the respective amino acid sequence it translates to. a, In the wild-type *RAB3B*sequence, a protospaceradjacent motif (PAM) and guide RNA sequences are represented. b, *RAB3B*sequence with heterogeneous mutation and resulting truncated amino acid sequence are represented. **Supplementary Figure S2.** Genome view of edited *RAB3B*. Genomic DNA from SK-HEP-1 (upper panel) and its derivative, RAB3B-KD cells (lower panel) were subjected to whole exome sequence using the TruSeqRapid Exome Library Prep Kit and NextSeq500 (Illumina). the filtered short reads were mapped to the reference genome (hg19) with BWA (version 0.7.12). The represented image was generated using IGV (version 2.9.4). The RAB3B-KD cells showed reads harboring insertion “T” at chromosome 1: 52,442,589 with 37% (11/30 reads) frequency. Mutation analysis using Strelka(version 0.4.10.2) showed no insertion/deletion variation by the off-target effect of the genome-editing. **Supplementary Figure S3.** Schematic for identification of genes specific for cancer stem-like cells and HCCs with poor prognosis. On the left, the identification was started with SK-HEP-1 cells in the sphere inducing and control conditions. On the right, the identification started from HCC specimens with/without recurrence after surgery. The represented number of starting genes showed that the number of genes with sum of a fragment-count of all samples was more than half of the sample number. DEGs, differentially expressed genes with > 2-fold change, *q* < 0.05, and the average count in higher group > 50. **Supplementary Figure S4.** mRNA levels of the identified genes determined using RNA-seqanalysis. The mRNA levels of the five identified cancer stem-like cell specific upregulated genes in SK-HEP-1 cells (a) and clinical specimens (b) are represented as transcripts per million (TPM). **Supplementary Figure S5.** Cell cycle analysis. Cell cycle distribution of SK-HEP-1 (aand b), *RAB3B*-KO (cand d), and KD/pRAB3B (eand f) cells in control (a, c, and e) and sphere inducing (b, d, and f) conditions, respectively. After cultivation, cells were dissociated with Accumax(Innovative Cell Technologies, San Diego, CA, USA). Cell cycle distribution was analyzed by flow cytometry, following propidiumiodide (PI) staining. Cells were fixed with 70% ethanol and then resuspendedin PI/RNase Staining Buffer (BD Biosciences, Franklin Lakes, NJ). The DNA content of cells was analyzed using a MACSQuantanalyzer (MiltenyiBiotec, BergischGladbach, Germany). Each panel shows a representative histogram. **Supplementary Figure S6.** BCAN9370Liver metastasis ability of RAB3B-KD cells. The metastatic ability of cells was evaluated by splenic injection into NOD-Rag1^null^IL2rγ^null^double mutant mice. Representative images in Table 1, mice were injected with 1 x 10^4^ tumor cells. RAB3B-KD is a *RAB3B*-edited clone derived from SK-HEP-1 cells. Yellow arrows indicate formed tumors. **Supplementary Figure S7.** Schematic for identification of *RAB3B*-affected cancer stem-like cell (CSLC) specific genes. With SK-HEP-1 derivative cells; parental SK-HEP-1, *RAB3B*-KD, and KD/pRAB3B, *RAB3B*affected CSLC specific differentially expressed genes (DEGs) were identified using RNA-seqanalysis. On the left, the DEGs in sphere-inducing conditions were identified using the same criteria as mentioned in Supplementary Fig. S3. On the right, *RAB3B*-affected genes in control conditions were identified as DEGs with > 1.7-fold change, *q* < 0.05, and average count in higher group > 30. The circled numbers are the screening criteria as shown in the text. The represented number of starting genes shows that the number of genes with sum of a fragment-count of all samples was more than half of the sample number. **Supplementary Figure S8.** Full-length blots used in Fig. 2. **Supplementary Figure S9**. Full-length blots at different exposure time used in Fig. 2.

## Data Availability

The datasets used and/or analyzed during the current study are available from the corresponding author on reasonable request. RNA sequencing data of this study have been deposited in the DDBJ Sequenced Read Archive repository (https://www.ddbj.nig.ac.jp/index.html) with accession numbers DRA012980, DRA012981, and DRA012982.
