# Supplementary material for: Elevated expression of RAB3B plays important roles in chemoresistance and metastatic potential of hepatoma cells
Source: BMC Cancer. 2022 Mar 11;22:260. doi: 10.1186/s12885-022-09370-1 (PMC8917729; doi:10.1186/s12885-022-09370-1)
Supplement: Supplementary file 1 — Additional file 1: Table S1. Primers and hydrolysis probes used in this study. Supplementary Figure S1. Genome editing of RAB3B. In each panel, upper and lower figures show Sanger sequencing data and the respective amino acid sequence it translates to. a, In the wild-type RAB3Bsequence, a protospaceradjacent motif (PAM) and guide RNA sequences are represented. b, RAB3Bsequence with heterogeneous mutation and resulting truncated amino acid sequence are represented. Supplementary Figure S2. Genome view of edited RAB3B. Genomic DNA from SK-HEP-1 (upper panel) and its derivative, RAB3B-KD cells (lower panel) were subjected to whole exome sequence using the TruSeqRapid Exome Library Prep Kit and NextSeq500 (Illumina). the filtered short reads were mapped to the reference genome (hg19) with BWA (version 0.7.12). The represented image was generated using IGV (version 2.9.4). The RAB3B-KD cells showed reads harboring insertion “T” at chromosome 1: 52,442,589 with 37% (11/30 reads) frequency. Mutation analysis using Strelka(version 0.4.10.2) showed no insertion/deletion variation by the off-target effect of the genome-editing. Supplementary Figure S3. Schematic for identification of genes specific for cancer stem-like cells and HCCs with poor prognosis. On the left, the identification was started with SK-HEP-1 cells in the sphere inducing and control conditions. On the right, the identification started from HCC specimens with/without recurrence after surgery. The represented number of starting genes showed that the number of genes with sum of a fragment-count of all samples was more than half of the sample number. DEGs, differentially expressed genes with > 2-fold change, q < 0.05, and the average count in higher group > 50. Supplementary Figure S4. mRNA levels of the identified genes determined using RNA-seqanalysis. The mRNA levels of the five identified cancer stem-like cell specific upregulated genes in SK-HEP-1 cells (a) and clinical specimens (b) are represented as tra [file 12885_2022_9370_MOESM1_ESM.pdf]

Table S1 Primers and hydrolysis probes used in this study

Gene	Sequence
RAB3B	
5'-primer	5'-GGTTGTTCCCACTGAGAAGG-3'
3'-primer	5'-TTGCACTGGCTTCAAAGAAA-3'
Hydrolysis probe	UPL Probe #17*
ABCG2	
5'-primer	5'-TTCCACGATATGGATTTACGG-3'
3'-primer	5'-GTTTCCTGTTGCATTGAGTCC-3'
Hydrolysis probe	UPL Probe #29*
APOE	
5'-primer	5'-GGTCGCTTTTGGGATTACCT-3'
3'-primer	5'-CATGGTCTCGTCCATCAGC-3'
Hydrolysis probe	UPL Probe #17*
LEPR	
5'-primer	5'-CATCAGTGACATGTGGTCCTCT-3'
3'-primer	5'-ACCCTCAGCCTCAGAGAAGTT-3'
Hydrolysis probe	none**
LNX	
5'-primer	5'-ACTGTCAAGCAAGTGCAAAGAA-3'
3'-primer	5'-CTTCCTTTGGCAGACGGCTA-3'
Hydrolysis probe	none**
TSPAN13	
5'-primer	5'-GCCATGTGCTCCAATCATAG-3'
3'-primer	5'-GCCAAACACCCAGGATCTC-3'
Hydrolysis probe	UPL Probe #67*
GAPDH	
5'-primer	5'-AGCCACATCGCTCAGACAC-3'
3'-primer	5'-GCCCAATACGACCAAATCC-3'
Hydrolysis probe	UPL Probe #60*
PGK1	
5'-primer	5'-CTGTGGCTTCTGGCATACCT-3'
3'-primer	5'-CGAGTGACAGCCTCAGCATA-3'
Hydrolysis probe	UPL Probe #42*

*The number for the Universal ProbeLibrary probes (Roche Diagnostics, Tokyo, Japan).

**SYBR Green I was used instead of the hydrolysis probe.

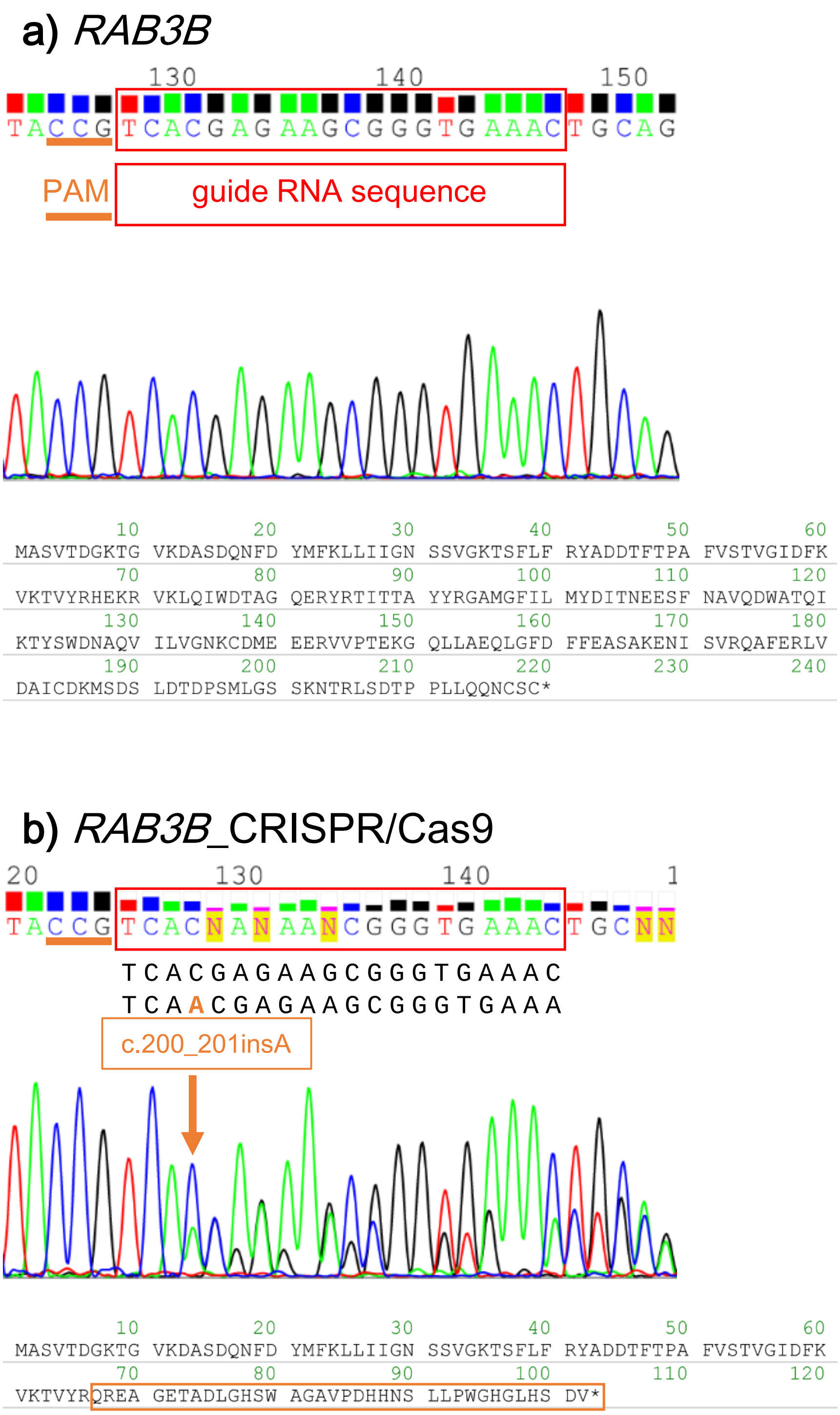

Supplementary Fig. S1 Genome editing of *RAB3B*. In each panel, upper and lower figures show Sanger sequencing data and the respective amino acid sequence it translates to. **a**, In the wild-type *RAB3B* sequence, a protospacer adjacent motif (PAM) and guide RNA sequences are represented. **b**, *RAB3B* sequence with heterogeneous mutation and resulting truncated amino acid sequence are represented.

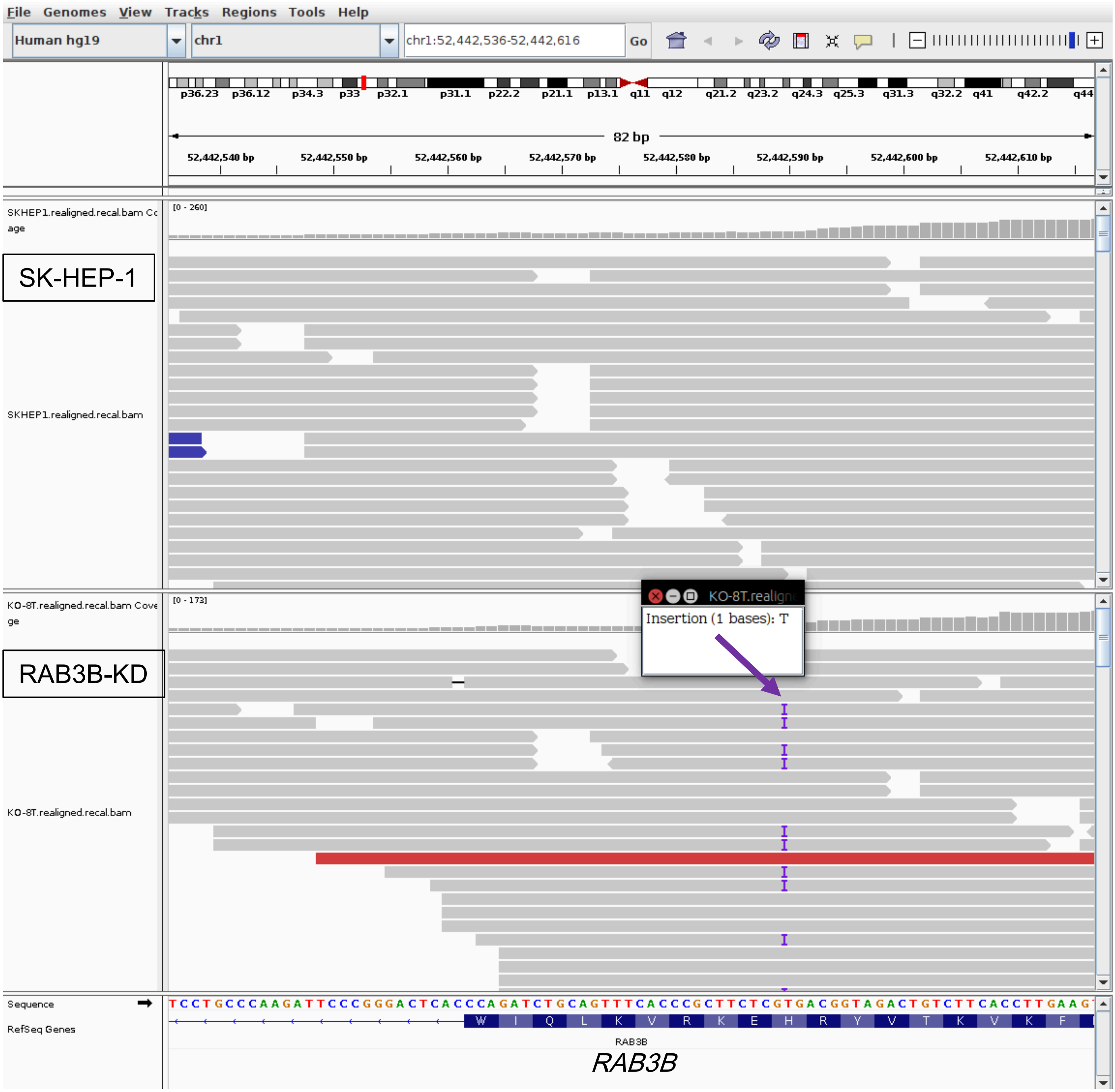

Supplementary Fig. S2 Genome view of edited *RAB3B*. Genomic DNA from SK-HEP-1 (upper panel) and its derivative, RAB3B-KD cells (lower panel) were subjected to whole exome sequence using the TruSeq Rapid Exome Library Prep Kit and NextSeq 500 (Illumina). the filtered short reads were mapped to the reference genome (hg19) with BWA (version 0.7.12). The represented image was generated using IGV (version 2.9.4). The RAB3B-KD cells showed reads harboring insertion “T” at chromosome 1: 52,442,589 with 37% (11/30 reads) frequency. Mutation analysis using Strelka (version 0.4.10.2) showed no insertion/deletion variation by the off-target effect of the genome-editing.

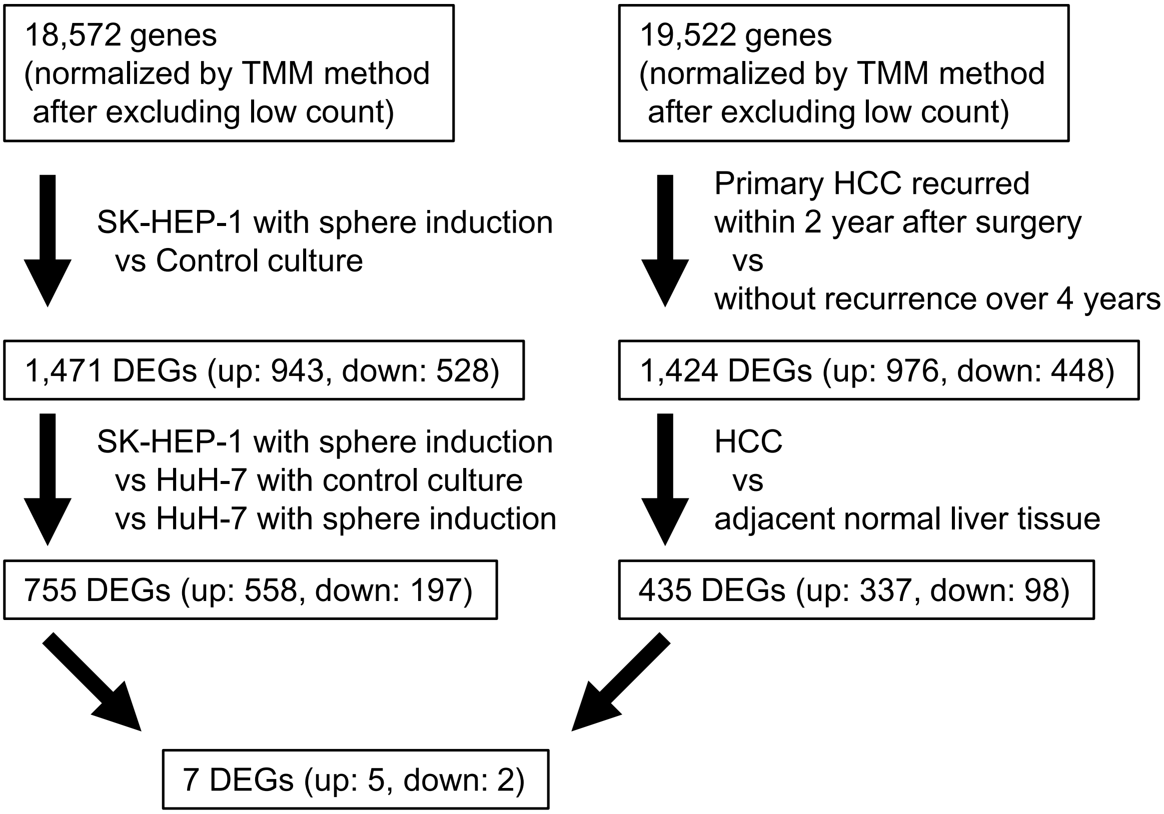

Supplementary Fig. S3 Schematic for identification of genes specific for cancer stem-like cells and HCCs with poor prognosis. On the left, the identification was started with SK-HEP-1 cells in the sphere inducing and control conditions. On the right, the identification started from HCC specimens with/without recurrence after surgery. The represented number of starting genes showed that the number of genes with sum of a fragment-count of all samples was more than half of the sample number. DEGs, differentially expressed genes with >2-fold change, $q < 0.05$, and the average count in higher group >50.

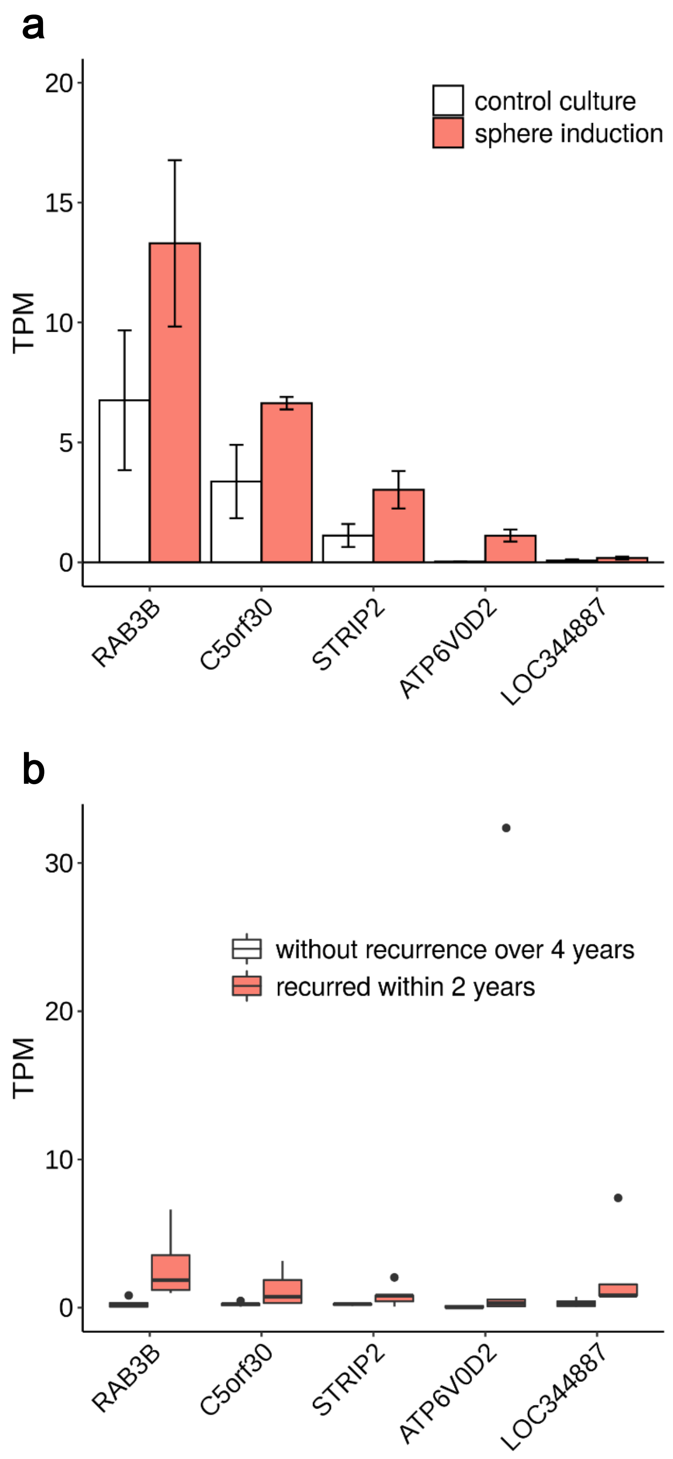

Supplementary Fig S4 mRNA levels of the identified genes determined using RNA-seq analysis. The mRNA levels of the five identified cancer stem-like cell specific upregulated genes in SK-HEP-1 cells (**a**) and clinical specimens (**b**) are represented as transcripts per million (TPM).

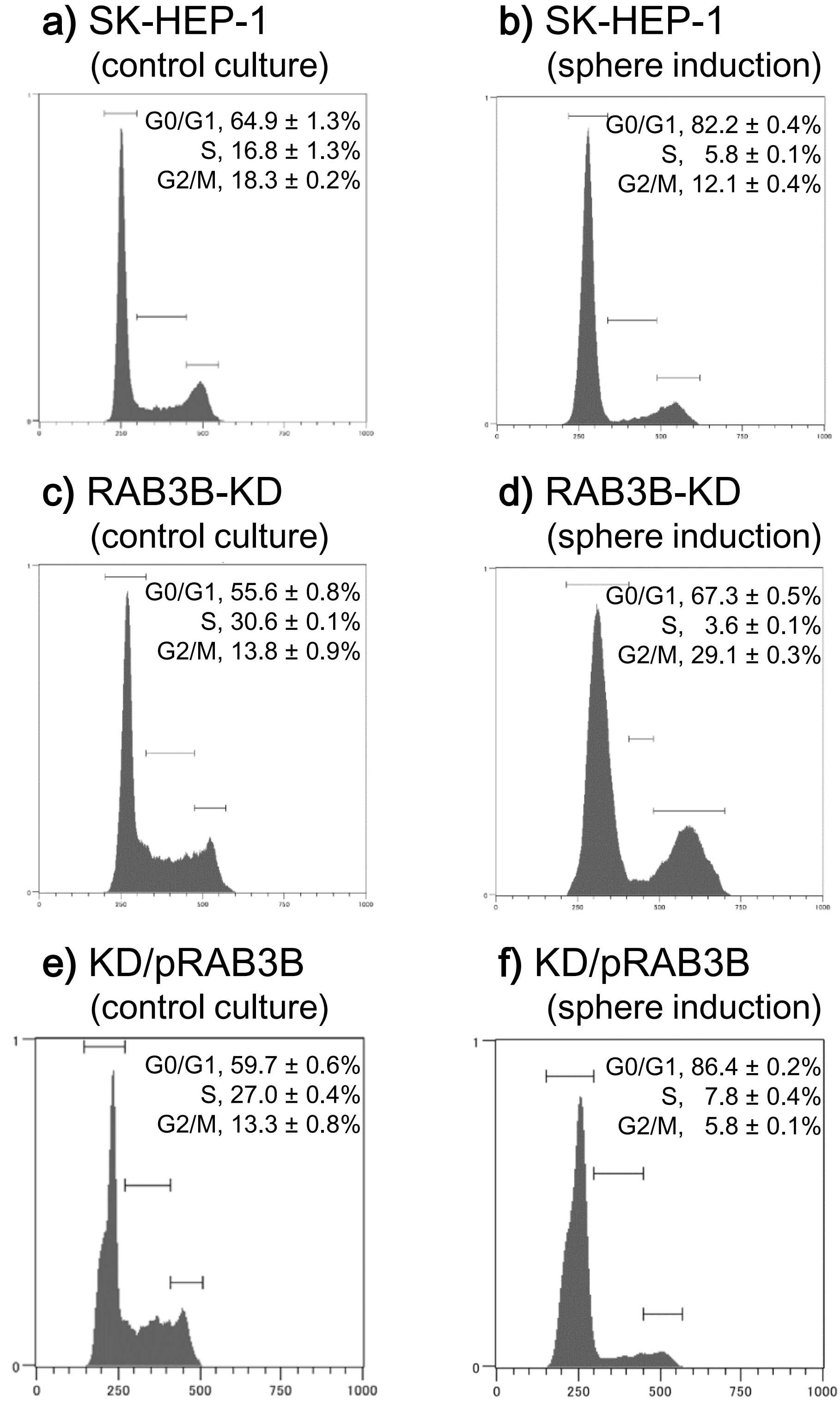

Supplementary Fig S5 Cell cycle analysis. Cell cycle distribution of SK-HEP-1 (**a** and **b**), *RAB3B*-KO (**c** and **d**), and KD/pRAB3B (**e** and **f**) cells in control (**a**, **c**, and **e**) and sphere inducing (**b**, **d**, and **f**) conditions, respectively. After cultivation, cells were dissociated with Accumax (Innovative Cell Technologies, San Diego, CA, USA). Cell cycle distribution was analyzed by flow cytometry, following propidium iodide (PI) staining. Cells were fixed with 70% ethanol and then resuspended in PI/RNase Staining Buffer (BD Biosciences, Franklin Lakes, NJ). The DNA content of cells was analyzed using a MACSQuant analyzer (Miltenyi Biotec, Bergisch Gladbach, Germany). Each panel shows a representative histogram.

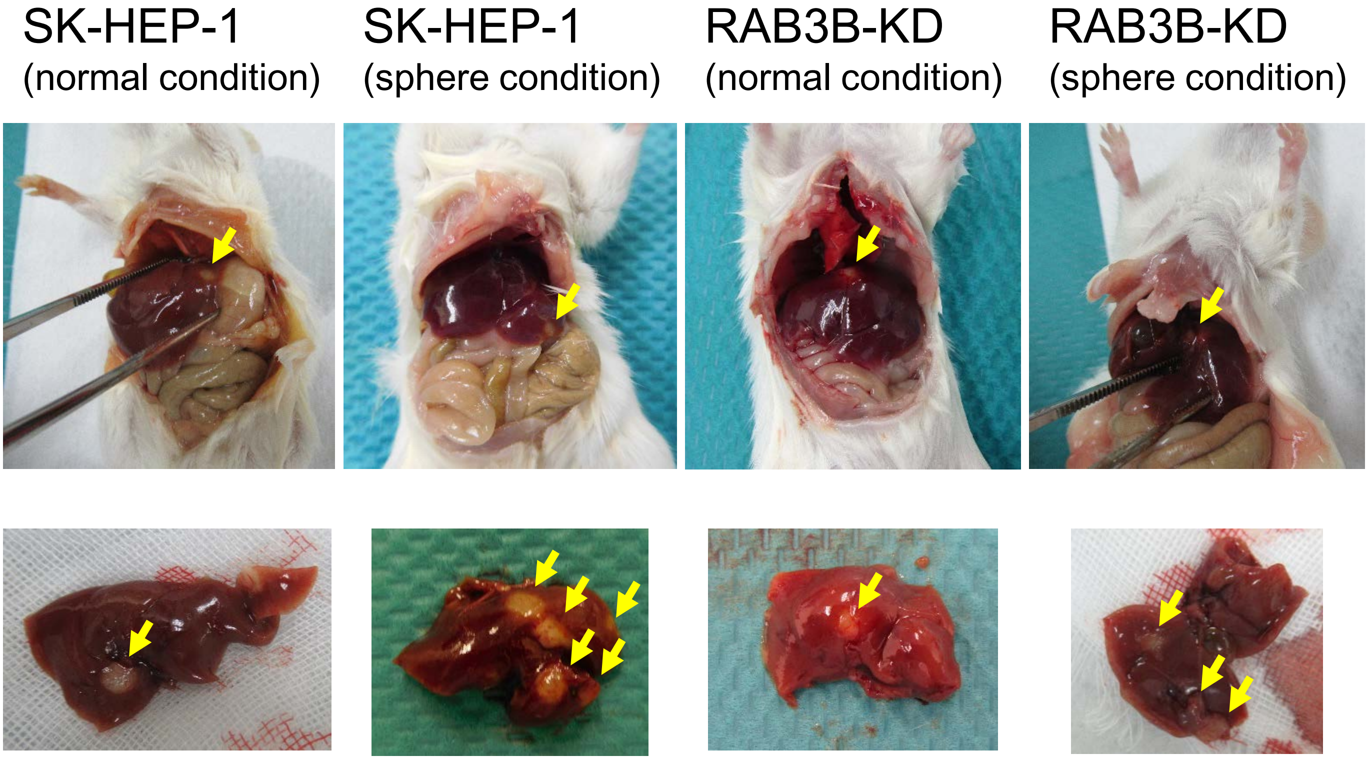

Supplementary Fig. S6 Liver metastasis ability of RAB3B-KD cells. The metastatic ability of cells was evaluated by splenic injection into NOD-Rag1^{null} IL2rγ^{null} double mutant mice. Representative images in Table 1, mice were injected with 1 x 10^4 tumor cells. RAB3B-KD is a *RAB3B*-edited clone derived from SK-HEP-1 cells. Yellow arrows indicate formed tumors.

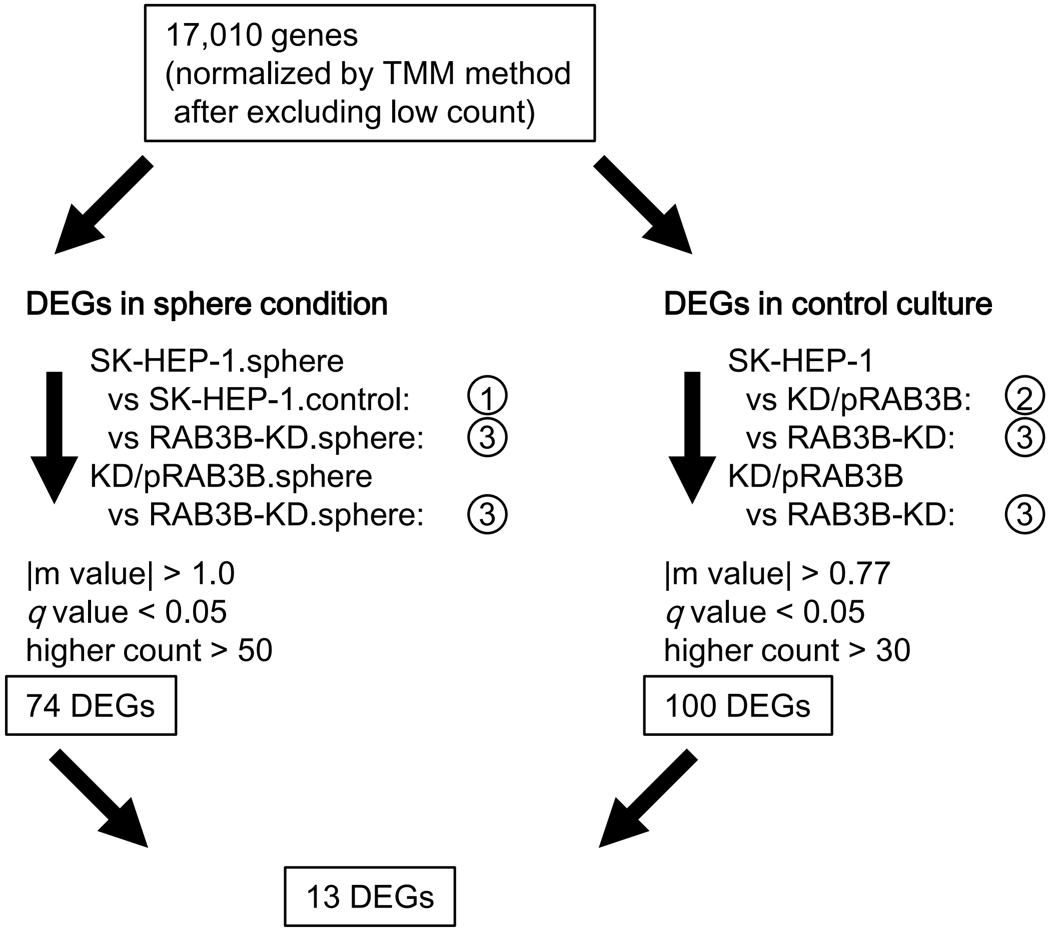

Supplementary Fig. S7 Schematic for identification of *RAB3B*-affected cancer stem-like cell (CSLC) specific genes. With SK-HEP-1 derivative cells; parental SK-HEP-1, *RAB3B*-KD, and KD/pRAB3B, *RAB3B* affected CSLC specific differentially expressed genes (DEGs) were identified using RNA-seq analysis. On the left, the DEGs in sphere-inducing conditions were identified using the same criteria as mentioned in Supplementary Fig. S3. On the right, *RAB3B*-affected genes in control conditions were identified as DEGs with >1.7-fold change, $q < 0.05$, and average count in higher group >30. The circled numbers are the screening criteria as shown in the text. The represented number of starting genes shows that the number of genes with sum of a fragment-count of all samples was more than half of the sample number.

RAB3B (target protein)

Fig. 2b left

1 2 3 4 5 6 1’ 2’ 3’ 5’ 4’ 6’

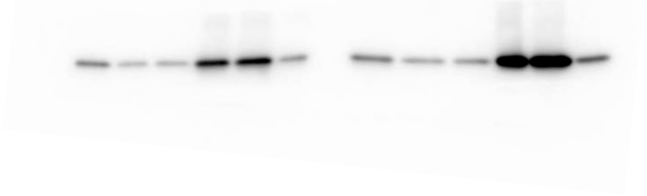

VCP (internal control)

Fig. 2b left

1 2 3 4 5 6 1’ 2’ 3’ 5’ 4’ 6’

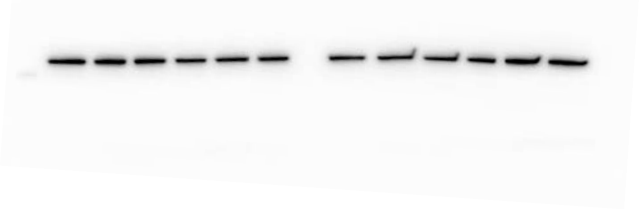

Lane 1, RAB3B-KD in normal condition.
Lane 2, RAB3B-KD in sphere induced condition.
Lane 3, SK-HEP1 in normal condition.
Lane 4, SK-HEP1 in sphere induce condition.
Lane 5, SK-HEP1 in another condition not related to this study.
Lane 6, SK-HEP1 in another condition not related to this study.
Lanes 1-6 and 1'-6' are the same sample, in a slightly different order.
Lane 1-4 were used in Figure 2b left panel.

RAB3B (target protein)

Fig. 2b right

1 2 3 4 5 6 7 8 1’ 2’ 9

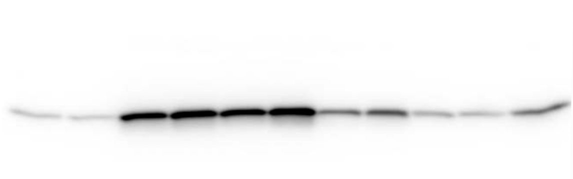

VCP (internal control)

Fig. 2b right

1 2 3 4 5 6 7 8 1’ 2’ 9

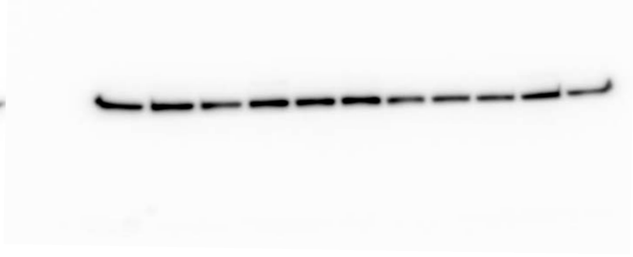

Lane 1, RAB3B-KD in normal condition.
Lane 2, RAB3B-KD in sphere induced condition.
Lane 3, RAB3B-KD/pRAB3B in normal condition.
Lane 4, RAB3B-KD/pRAB3B induce condition.
Lane 5-9, Another cell lysate not related to this study.
Lanes 1-2 and 1'-2' are the same sample.
Lane 1-4 were used in Figure 2b right panel.

Supplementary Fig. S8 Full-length blots used in Figure 2.

RAB3B (target protein)

Fig. 2b left

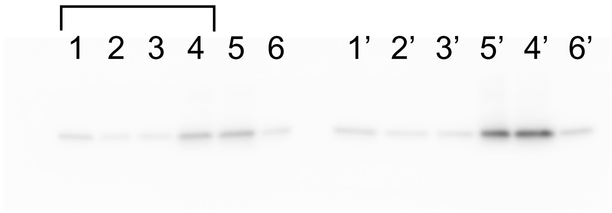

VCP (internal control)

Fig. 2b left

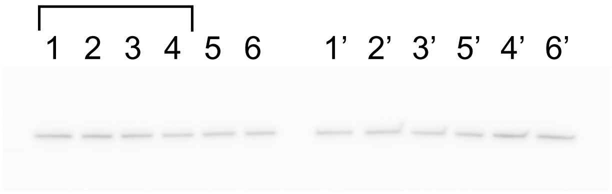

Lane 1, RAB3B-KD in normal condition.
Lane 2, RAB3B-KD in sphere induced condition.
Lane 3, SK-HEP1 in normal condition.
Lane 4, SK-HEP1 in sphere induce condition.
Lane 5, SK-HEP1 in another condition not related to this study.
Lane 6, SK-HEP1 in another condition not related to this study.
Lanes 1-6 and 1'-6' are the same sample, in a slightly different order.
Lane 1-4 were used in Figure 2b left panel.

RAB3B (target protein)

Fig. 2b right

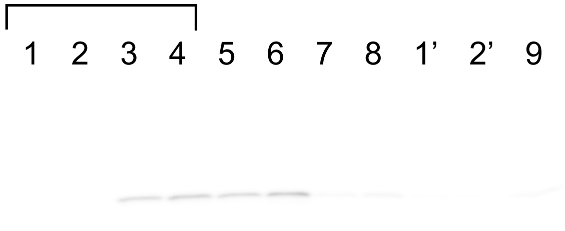

VCP (internal control)

Fig. 2b right

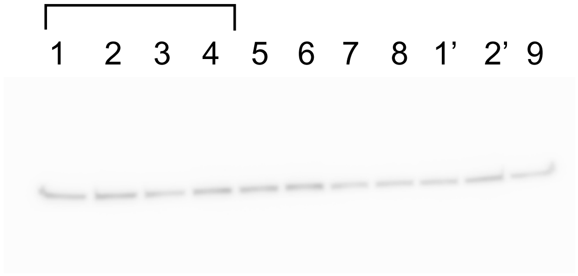

Lane 1, RAB3B-KD in normal condition.
Lane 2, RAB3B-KD in sphere induced condition.
Lane 3, RAB3B-KD/pRAB3B in normal condition.
Lane 4, RAB3B-KD/pRAB3B induce condition.
Lane 5-9, Another cell lysate not related to this study.
Lanes 1-2 and 1'-2' are the same sample.
Lane 1-4 were used in Figure 2b right panel.

Supplementary Fig. S9 Full-length blots at different exposure time used in Figure 2.
